# GABA receptor antagonism elicits feeding in the septohypothalamic nucleus

**DOI:** 10.3389/fnbeh.2025.1633659

**Published:** 2025-08-04

**Authors:** Ivett Gabriella, Vandana Nambiar, Chlinton Kuang, Abinanda Mukundan, Jonathan Dang, Aneerudh Venkatraghavan, B. Glenn Stanley

**Affiliations:** ^1^Stanley Laboratory, Psychology Department, University of California, Riverside, Riverside, CA, United States; ^2^Stanley Laboratory, Department of Molecular, Cell and Systems Biology, University of California, Riverside, Riverside, CA, United States

**Keywords:** septohypothalamic nuclei, GABA receptors, feeding, behavioral responses, picrotoxin, bicuculline, saclofen

## Abstract

**Introduction:**

Current rates of obesity and eating disorders have been steadily increasing, highlighting the importance of understanding the neural circuits of eating. This study explores the potential role of an understudied brain region, the septohypothalamic nucleus (SHy), in feeding control. Based on a serendipitous observation, we hypothesized that central injections of gamma-aminobutyric acid (GABA) receptor antagonists in the SHy would elicit feeding.

**Method:**

Adult male Sprague-Dawley rats (*n* = 39) were microinjected with a vehicle or GABA_A_ receptor antagonists (bicuculline or picrotoxin) or a GABA_B_ receptor antagonist (2-(S)-(+)-2-hydroxy-saclofen [2-OH saclofen]). Food and water intakes were measured at 1, 2, 3, and 24 h after injection, and behavioral responses (sleeping, resting, locomotor activity, vigorous activity, and grooming) were measured for 1 h.

**Result:**

Results showed increased food intake after bicuculline (*p* < 0.001) and picrotoxin (*p* = 0.03) injections during the 2nd and 3rd hours compared to controls. In addition, we found increased food intake 1 hour after 2-OH saclofen injections (*p* < 0.001). As for other behaviors, all three of the drugs suppressed resting (bicuculline: *p* < 0.001; picrotoxin: *p* < 0.001; 2-OH saclofen: *p* < 0.01) and increased locomotor activity (bicuculline: *p* < 0.001; picrotoxin: *p* < 0.001; 2-OH saclofen: *p* = 0.02).

**Discussion:**

Our findings suggest that GABA_A_ or GABA_B_ receptor deactivation by antagonists elicited eating with a delayed effect and increased general arousal in rats. These findings collectively suggest that SHy neurons expressing GABA_A_ and/or GABA_B_ receptors are elements of a neurocircuit that participates in the regulation of feeding.

## 1 Introduction

Worldwide, 2.5 billion adults (18 and older) were considered overweight in 2022 (World Health Organization, 2025). This number has been steadily increasing in the past decades (Dragano et al., 2020; Emmerich et al., 2024). Similarly, the rates of eating disorders have also been on the rise (Qian et al., 2021). These public health concerns give grounds for more research about the neural underpinnings of eating. Understanding the brain mechanisms of eating may help in prevention and treatment efforts.

The septohypothalamic nucleus (SHy) is located in the anterior portion of the hypothalamus; it borders the median preoptic nucleus, the medial and the lateral septum, the bed nucleus of the stria terminalis, and is near the nucleus accumbens. To our knowledge, no studies have directly examined the SHy brain region in relation to eating. However, serendipitously generated data by our lab suggested such a role for the SHy. We previously studied the role of gamma-aminobutyric acid (GABA) receptors in the lateral septum in relation to feeding (Calderwood et al., 2020; Gabriella et al., 2022). During our study, we noticed unexpected eating responses from a few of the rats in the sample. Specifically, eating was induced after the injection of a GABA antagonist in a small subset of subjects, while it reduced feeding in the rest of the subjects. After completing the histological evaluation, we learned that all four of the rats that responded this way had a misplaced cannula in the SHy instead of the lateral septum. This observation prompted our lab to investigate the possibility that the SHy brain region is involved in feeding.

The SHy projects to several different nuclei of the hypothalamus, some of which are important in eating mechanisms. Based on Chiba and Murata (1985), it has bidirectional connections to the medial preoptic nucleus and the periventricular area of the hypothalamus in the rat. Ugur et al. (2021) showed moderate projections to the subfornical lateral hypothalamus in the mouse brain, and Barbier et al. (2021) found projections from the SHy to POMC and AGRP neurons in the arcuate nucleus of the hypothalamus in mice. In addition, Jirikowski et al. (1988) showed projections to the lateral septum in the rat brain, which is also part of the neural circuit of eating.

Moreover, the SHy receives projections from many nearby nuclei that are involved in eating. For example, Zhu et al. (2024) showed projections from Phenylethanolamine N-methyltransferase-expressing neurons in the nucleus tractus solitarii using a modified rabies virus-based retrograde tracing method, and Nasanbuyan et al. (2025) showed projections from the oxytocin receptor-expressing neurons of the ventrolateral part of the ventromedial hypothalamus using viral vectors in mice. Shin and Ikemoto (2010) showed c-Fos activation in both the SHy and the septal area of rats after injecting picrotoxin, a GABA_A_ receptor antagonist, into the supramammillary nucleus of the hypothalamus, which indicates an inhibitory projection to the SHy. Based on anterograde viral tracing, the rostroventral lateral septum has also been shown to send inhibitory projections to the SHy in rats (Yeates et al., 2022). The SHy was also shown to be activated after muscimol injection in the nucleus accumbens (Stratford, 2005), revealing the potential for another inhibitory projection. In addition, after optogenetic stimulation of the periaqueductal gray area, PET images demonstrated that glucose metabolism increased in the SHy of the rat, indicating a downstream placement (He et al., 2019). These connections of the SHy to major eating centers, such as the hypothalamus, septum, and the nucleus accumbens, point to a potential role for the SHy in feeding.

GABA is the most widespread inhibitory neurotransmitter in the brain (Ghit et al., 2021) and primarily binds to two different receptor types. GABA_A_ receptors are fast-acting ionotropic receptors, while GABA_B_ receptors are metabotropic and have a much slower action (Wang et al., 2024). Both GABA_A_ and GABA_B_ receptors have been implicated in feeding mechanisms in brain regions that connect to the SHy. For example, activation of GABA_A_ receptors promotes satiety in the lateral hypothalamus (Stanley et al., 2011; Turenius et al., 2009); while they promote feeding in the paraventricular and the ventromedial hypothalamus (Wang et al., 2024). In addition, activation of either GABA receptor types induces rapid feeding in the lateral septum (Gabriella et al., 2022) and the nucleus accumbens (Marinescu and Labouesse, 2024; Stratford and Kelley, 1997). Based on these studies, GABA receptors are important modulators of feeding; if they are present in the SHy, they may be involved in these mechanisms.

While the types of neurons and receptors of the SHy have not been thoroughly investigated, a few studies included information about them. Rao et al. (1987) showed that the rat SHy contains some cholinergic neurons, and Okamura et al. (1990) found indications that it also contains GABAergic neurons. In addition, Rogers Flattery et al. (2021) employed receptor autoradiography and found dense oxytocin and vasopressin receptors in the SHy of chimpanzees. Neurotensin receptors were found by Najimi et al. (2014), and mu opioid receptors were shown by Mansour et al. (1994). To our current knowledge, no study has involved GABA receptors in the SHy. However, our accidental findings indicated the presence of GABA receptors. Therefore, we hypothesized that the SHy would also include receptors for GABA, which may be involved in feeding mechanisms.

A role for the SHy in feeding has not previously been directly investigated. However, a few studies included this brain region within their findings, supporting further investigation. In particular, Nakahara et al. (2004) found that c-Fos expression was increased in food-restricted rats during the feeding hours but not after the restricted hours, suggesting a potential association with eating but not anticipation of eating. Csikós et al. (2020) found that intraperitoneal injection of Deoxynivalenol, a mycotoxin, moderately increased c-Fos expression in GABAergic neurons in the SHy and reduced feeding. Similarly, Kakall et al. (2021) found moderate c-Fos expression in the SHy after 48 h, 5 days, and 14 days of a high-fat diet in the mouse brain, but there was no c-Fos increase in the regular chow feeding or the fasting groups. Additionally, Fos protein expression was shown in the ipsilateral SHy after muscimol injection in the nucleus accumbens shell. Since chemical inhibition of the nucleus accumbens shell is associated with elicited feeding in rats, the SHy may be a downstream projection involved in the nucleus accumbens shell's feeding circuit (Stratford, 2005). Although these studies did not directly implicate the SHy as an element of an eating control circuit, they do provide tangential evidence and suggest the need to investigate and resolve this issue.

The specific behavioral function of the SHy has not yet been elucidated, but a few studies across different species indicate diverse associations. More specifically, it may be involved with emotional processes, such as stress, fear, social defeat, affiliative behavior, and bodily functions, such as thermoregulation. Campeau et al. (1997) found increased brain activity in the SHy nucleus of male Sprague–Dawley rats by analyzing c-fos mRNA induction after the experience of stress due to loud sound (90–105 dBA). Similarly, research in other species has also shown involvement with emotionally negative stimuli. Day et al. (2004) found induced c-fos mRNA in the SHy nucleus after rats were presented with a fearful odor from fox feces, and Nasanbuyan et al. (2025) found c-Fos protein expression in the SHy after a social defeat paradigm in mice. Emotionally positive feelings, such as affiliation, have also been examined in multiple species. Gentle stroking of female rats decreased activity in the SHy and induced affiliative behavior toward humans (Okabe et al., 2021). Based on fMRI results, Moll et al. (2012) and Bortolini et al. (2021) implicated the SHy in social attachment and affiliative behavior in the human brain. In other studies, it has been shown to be activated by thermoregulatory processes (Bratincsak and Palkovits, 2004), and c-Fos expression has been shown to increase after treadmill running and an increase in body temperature in male Wistar rats (Lima et al., 2019). Collectively, these studies reveal various emotional and visceral functions associated with the SHy. Based on this information, we aimed to further investigate what types of behaviors might be associated with GABA receptors in addition to feeding.

The aim of this study was to explore the role of the SHy in eating and general behavioral responses (sleeping, resting, locomotor activity, vigorous activity, and grooming). We hypothesized that the SHy contains GABA receptors and that central injections of GABA_A_ and GABA_B_ antagonists (bicuculline, picrotoxin, and 2-hydroxysaclofen) would modulate feeding. In addition, we hypothesized that if GABA is inhibitory in this area, then general behavioral activity levels would increase due to GABA receptor antagonism. On the other hand, if GABA's role is disinhibitory, then activity levels would decrease. To our knowledge, this study is the first to investigate the role of the SHy in eating and behavioral responses.

## 2 Materials and methods

### 2.1 Animals

These experiments employed adult male Sprague–Dawley rats (Charles River Laboratories, Wilmington, MA, USA) weighing 350 to 400 g at the time of the surgery. Rats were ordered before each experiment and were allowed to acclimate for 1 week before the surgeries. All rats were individually housed in a temperature-controlled vivarium on a 12:12-h light-dark cycle. All the experiments conducted were in compliance with the regulations set by the Institutional Animal Care and Use Committee at the University of California, Riverside.

### 2.2 Surgical procedures

Surgeries were performed on all rats to permanently implant cannulas terminating in the SHy. All rats received acetaminophen (Children's Tylenol and drinking water, 1 mg/ml) as an analgesic, dissolved in their drinking water 1 day before and 2 days after the surgery, which they could self-administer *ad libitum*. Animals were injected just before surgery with 0.4 ml of atropine sulfate (0.54 mg/ml) to reduce salivation and bronchial secretions. Then, they were anesthetized by intraperitoneal injections of Ketamine HCl (80 mg/ml) at 1 ml/kg of body weight and Xylazine HCl (0.02 ml). One stainless-steel guide cannula (26 gauge, 18 mm long) was stereotaxically implanted 1 mm dorsal to the SHy target on the right side of the animals. The coordinates used for the SHy were 9.0 mm anterior to the interaural line, 0.4 mm lateral to the midsagittal sinus, and 5.6 mm ventral to the surface of the skull, with the incisor bar 3.3 mm below the center of the earbars. Cannulas were held in place by four stainless-steel skull screws and dental acrylic. A plastic guard was embedded into the dental acrylic to protect the cannulas, which were sealed with a removable 33-gauge stainless-steel obturator. Rats were given at least seven days to recover from surgery, during which time they were handled and mock-injected multiple times to habituate them to the injection procedures.

### 2.3 Drugs

All drugs were purchased from Tocris Bioscience (Minneapolis, MN, USA). The injection volume in all experiments was 0.3 μl. In the first study, the GABA_A_ receptor antagonist bicuculline (1.5 μg/0.3 μl) was used as the experimental manipulation. Bicuculline is a competitive antagonist that blocks the binding of GABA to the GABA_A_ receptor. For optimal solubility, dimethyl sulfoxide (DMSO) was used to dissolve bicuculline, and DMSO was used as the vehicle in the experiments using bicuculline.

In the second study we used picrotoxin, another GABA_A_ antagonist that works by a different mechanism than bicuculline. The dose of 0.1 μg / 0.3 μl was chosen in the sub-seizure middle range based on our previous study involving the lateral septum (Gabriella et al., 2022). The vehicle in the experiments using picrotoxin was artificial cerebrospinal fluid (aCSF; 147 mM Na^+^, 154 mM Cl^–^, 3 mM K^+^, 1.2 mM Ca^2+^, and 0.09 mM Mg^2+^).

In the 3rd study, we used the competitive GABA_B_ antagonist 2-OH saclofen. We used the active enantiomer of saclofen, 2-(S)-(+)-2-hydroxy-saclofen (2-OH saclofen; 1.25 μg / 0.3 μl), which blocks the binding of GABA to GABA_B_ receptors. 2-OH saclofen was dissolved in distilled water for improved solubility, and aCSF was used as the vehicle.

### 2.4 Experimental design

The rats were provided with unlimited access to rat chow (5001 - Laboratory Rodent Diet) and water up until three days prior to testing. At that time, their regular rat chow was replaced with a highly palatable sweet milk mash diet, also available in unlimited quantities. This mash food was a blend of the rat chow (500 g), sugar (400 g), and 14 oz of condensed milk (Carnation). The palatable nature of the mash diet ensures that the rats consume adequate amounts of food without limiting intake due to taste aversion. Its moist texture minimized the need for water during chewing and digestion, reducing the rats' need to drink during meals. Importantly, the mash diet was introduced three days prior to the experiment, eliminating novelty effects and reducing the likelihood of overconsumption due to increased interest in a new food. In addition to the mash food, the rats received rat chow pellets between injection days as enrichment. To ensure the rats were satiated before testing, they were given fresh mash food one hour prior to receiving injections in all experiments.

**Figure 1:**
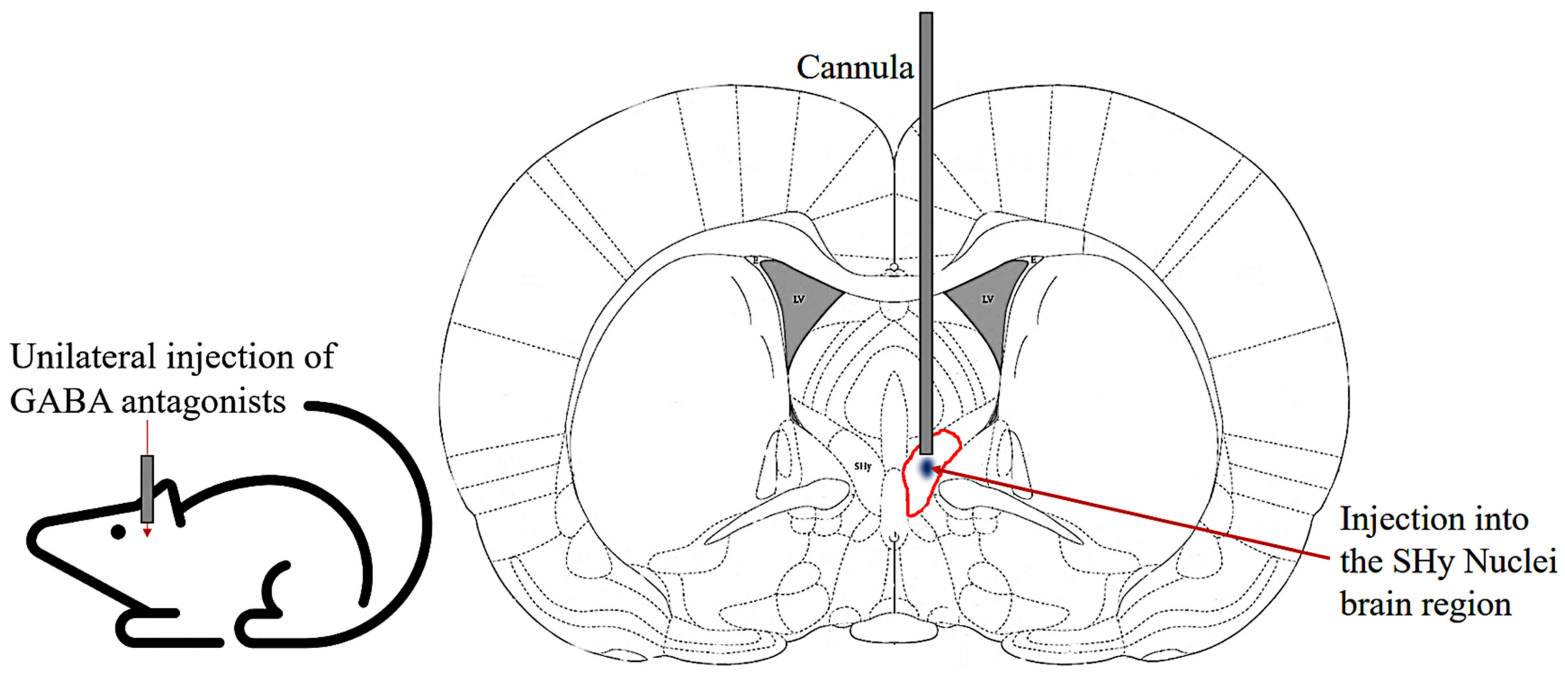
Schematic of cannula implanted into the SHy nucleus of the rat. Coronal section illustrating the unilateral injection of GABA antagonists into the SHy (circled in red) through chronically implanted guide cannulas.

As illustrated in Figure 1, the tests consisted of administering central injections of a vehicle, GABA_A_ receptor antagonists (bicuculline and or picrotoxin), or a GABA_B_ receptor antagonist (2-OH saclofen) into the SHy to observe their impact on eating behavior. The injections were administered using an injector that extended 1 mm past the cannula onto the SHy on the right side of the brain, with a 10-second pause before removing the injector. The study involved three groups of rats. One group received bicuculline, another picrotoxin, and a third one received 2-OH saclofen injections. The vehicle injections were administered on two different days to all rats. Because we expected more variability in the response to the drug compared to the vehicle, we administered the drug on three separate days to all rats to capture this variability. Therefore, all rats received a total of 5 injections in a counterbalanced design with a 1-day rest period in between injections. We measured the weight of the food bowl and the water bottle immediately before the first injection and at the 1, 2, 3, and 24 h mark post-injection. The difference in weight was calculated and constituted as the measured food intake in grams and the water intake in milliliters.

Additionally, we observed and coded the behavioral effects once per minute for 60 min following the injection. The observation involved a visual snapshot of what the rat was doing inside his home cage at that moment. The behavior was coded by two observers simultaneously. Each observer coded one half of the group, assessing a distinct subset of rats. As a result, each rat was only observed by a single observer. Observers were blind to the experimental condition of the rats they observed. The behaviors were categorized as sleeping (motionlessly laying on the floor), resting (motionlessly sitting on the floor with the head in an upright position), locomotor activity (the rat is upright on all four legs and displays light movement of the head or body), vigorous activity (walking up and down or rearing up on hind legs), and grooming (repetitive cleaning of the body and the face by licking and rubbing the paws) (Estanislau et al., 2019; Li et al., 2024).

### 2.5 Histological procedures

Once behavioral tests were concluded, the rats received an intracerebral microinjection of Chicago Sky Blue 6B (Alfa Aesar, Thermo Fisher Scientific, Waltham, MA, USA) stain to indicate the injection site. Following this, they were humanely euthanized with an overdose of sodium pentobarbital (0.4 ml; Fatal Plus, Vortech Pharmaceuticals, LTD, Dearborn, MI, USA) and transcardially perfused with a formalin solution containing 10% formaldehyde. Brains were extracted and stored in the same formalin solution. The fixed brains were sectioned into 60 μm coronal brain slices on a freezing microtome and Nissl stained with cresyl violet. The mounted brain sections were used to verify the injection sites, which were matched to the coordinates by Paxinos and Watson (2013).

### 2.6 Statistical analysis

Statistical analysis was conducted using IBM Statistical Product and Service Solutions (SPSS) 27 software. Food intake during the first 3 h was measured to assess the short-term effects of the drug. A two-way repeated-measures Analysis of Variance (ANOVA) was utilized to analyze the food and water intake at 1, 2, and 3 h. The data included two within-subject variables—Drug (2 levels) and Time (3 levels). To evaluate potential long-term effects, the 24-h food and water intake was assessed using a paired sample *t*-test; the 24-h measurements involved only two conditions (vehicle and drug). The data used was the average food and water intake across 2 days of vehicle and 3 days of drug injections for each rat. To assess the magnitude of behavioral effects and compare them across pharmacological conditions, a between-subject design was employed. Accordingly, a mixed-model ANOVA was conducted to analyze the behavioral data.

Before conducting these ANOVA tests, sphericity assumptions were verified through Mauchly's test (Mauchly, 1940). Greenhouse–Geisser correction (Greenhouse and Geisser, 1959) was applied to the results of the data that did not meet the assumptions. Significant ANOVA tests were followed by a *post-hoc* analysis, which involved paired-sample *t*-tests between each pair of variables at matched post-injection time points. Bonferroni correction (Bonferroni, 1936) was applied to all p-values to correct for Type I errors.

The initial sample consisted of 42 rats; 14 animals per three drug groups. Three rats were excluded from the sample due to unverifiable injection sites: one exhibited enlarged ventricles and apparent damage near the injection site, while histological processing resulted in tissue damage in the remaining two. Therefore, the analysis only included a total of 39 rats. Within these 39 rats, those subjects whose histological analysis indicated that the injection site was outside the SHy were re-categorized as an Off-Target Group (*n* = 8), and their data were analyzed separately. As a result, the final sample sizes were reduced as follows: the bicuculline group decreased from 14 to 9, the picrotoxin group from 14 to 12, and the 2-OH saclofen group from 14 to 10.

Vehicle injection data were pooled across conditions to avoid making comparisons against three separate vehicle groups (two aCSF and one DMSO). The pooled vehicle group was created from all the vehicle injection data from all three experiments and randomly sampled to have a relatively equal sample size (*n* = 12). We used an independent samples t-test to check mean differences between the DMSO and aCSF food intake at 1, 2, and 3 h. Although the chemical components of the vehicle injections varied among the different groups, they did not show statistically significant differences (Supplementary Table S1).

Data are presented as means with 1 standard error of the mean (SEM). Effect sizes are denoted as Partial Eta Squared ($\eta_{p}^{2}$). The “n” values represent the number of animals in each experiment. The histology, feeding, and behavioral data sets are available on the Open Science Framework website and can be accessed at https://osf.io/d5kur (Gabriella, 2025a). The website also includes the analysis code, supporting videos, and supplementary materials (Gabriella, 2025b).

## 3 Results

### 3.1 Histology

**Figure 2:**
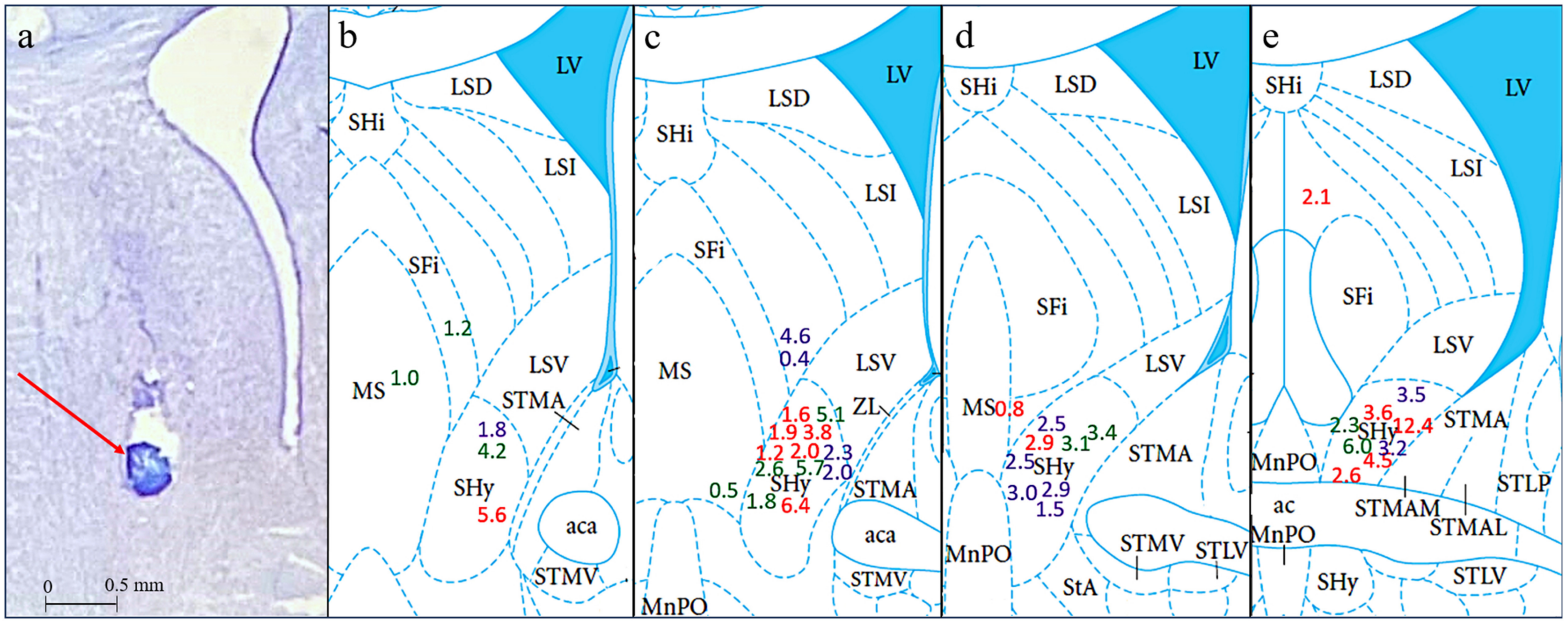
Histology results in coronal sections. **(a)** Is a cresyl violet stained section showing a representative injection site indicated by a red arrow and a scale bar of 0.5 mm. **(b–e)** Show interaural coordinates 9.2, 9.1, 9.0, and 8.9 mm, from (Paxinos and Watson, 2013). Colored numbers indicate the cumulative grams of food intake for each rat 3 h post-injection at the approximate injection sites inside or, in a few cases, outside the SHy. Green = Bicuculline; Red = Picrotoxin; Purple = 2-OH Saclofen.

Histology was performed after each experiment to match the location of the injections to the SHy target site. We had a total of 39 rats. The injection site was localized within the SHy in 9 rats in the bicuculline group, 12 rats in the picrotoxin group, and 10 rats in the 2-OH saclofen group, from 9.2 to 8.9 mm anterior to the interaural line (Figure 2). Inaccurate injection placements were observed in 8 rats. Specifically, the injection site did not match the intended SHy target in 3 rats that received bicuculline, 3 rats that received prcotoxin, and 2 rats that received 2-OH saclofen. These off-target injections are illustrated outside the SHy region in Figure 2, except for 1 rat in the picrotoxin group that had an injection at 8.6 mm AP—outside the target region—which is not depicted in the figure. All of these animals with off-target injections were grouped together into an *Off-target* group (*n* = 8). Rats that received Bicuculline injection are indicated by green, rats that received Picrotoxin are indicated by red, and rats that received 2-OH Saclofen are indicated by purple in the figures.

### 3.2 Eating and drinking results

#### 3.2.1 Bicuculline in the SHy elicits feeding

**Figure 3:**
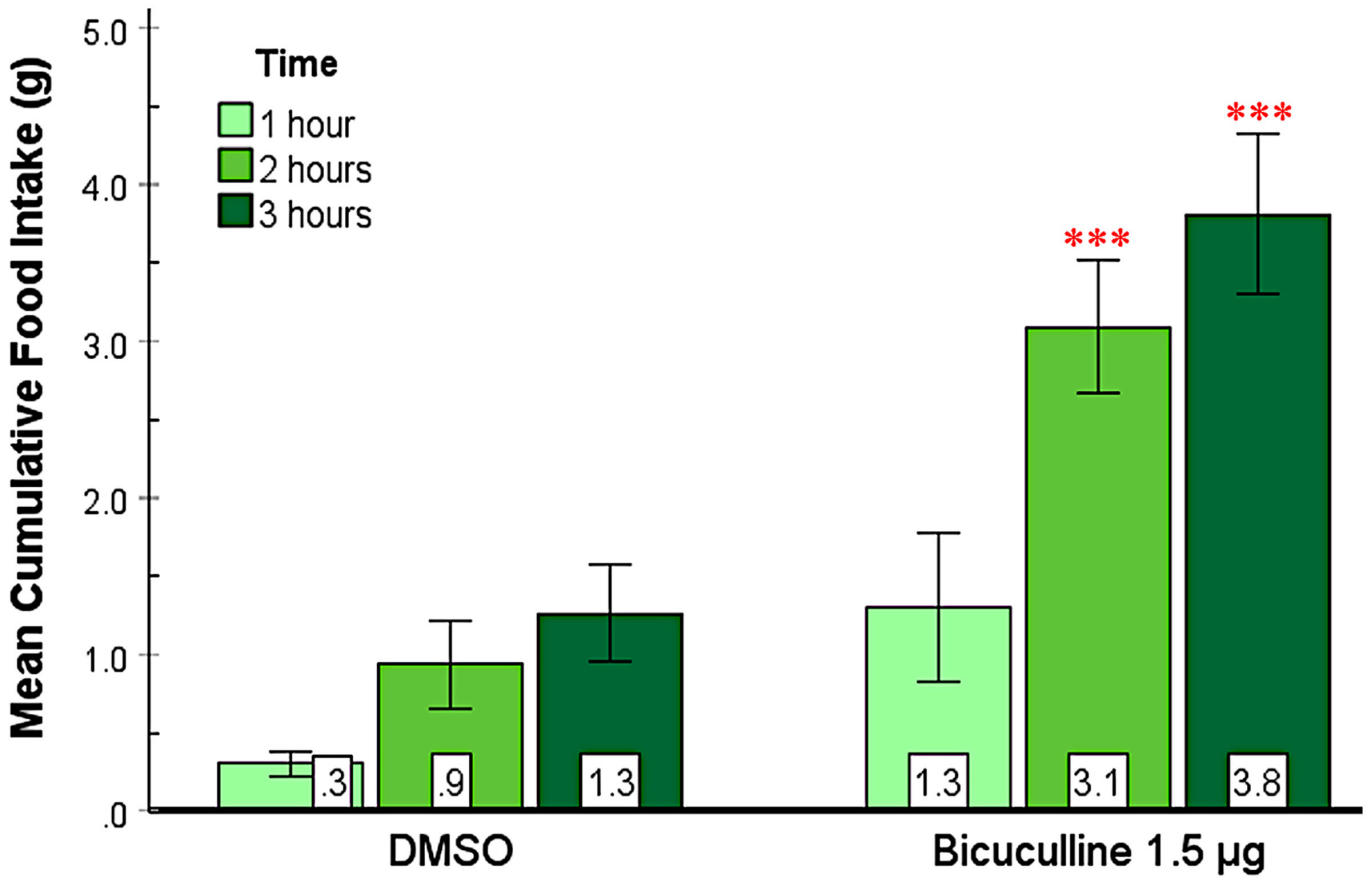
Bicuculline Injected into the SHy Elicited a Delayed Feeding Response. Error bars SEM; *n* = 9; mean values are shown at the bottom of each bar. *** indicates significant differences compared to the vehicle group at matched times at *p* < 0.001.

In this experiment, we examined the effect of bicuculline, a GABA_A_ receptor antagonist, on food and water intake in the SHy. As shown in Figure 3, bicuculline elicited consistent feeding in rats in the 2nd and 3rd h postinjection. A 2-way repeated measures ANOVA (*n* = 9) revealed significant main effects for both drugs [*F*_(1, 8)_ = 28.55, *p* < 0.001, $\eta_{p}^{2}=0.78$] and time [*F*_(1.1, 8.9)_ = 19.95, *p* = 0.001, $\eta_{p}^{2}=0.71$], and significant interaction [*F*_(1.2, 9.8)_ = 7.29, *p* = 0.02, $\eta_{p}^{2}=0.48$] between drugs and time. Based on the *post-hoc* analysis, bicuculline food intake was significantly larger compared to controls at 2 (*p* < 0.001) and 3 h (*p* < 0.001) post-injection but not during the first hour. Mean individual food intakes at 3 h are indicated in green in Figures 2b–e. These results indicate that bicuculline elicited a strong feeding response within 2 h post-injection. The 24-h food intake measurements did not reveal statistically significant differences (Supplementary Figure S1), suggesting that there were no long-term effects. Based on the water intake analysis, the main effects for both drugs [*F*_(1, 8)_ = 7.19, *p* = 0.03, $\eta_{p}^{2}=0.47$] and time [*F*_(1.1, 8.8)_ = 133.45, *p* < 0.001, $\eta_{p}^{2}=0.94$] were significant. However, there was no significant interaction. *Post hoc* analysis showed significantly increased drinking in the first (*p* = 0.02) and second hours (*p* = 0.03; Supplementary Figure S2) postinjection. In addition, the 24-h water intake also significantly increased in the bicuculline group, compared to controls (*t* = −1.96, *p* = 0.04, figure not shown). The elicited feeding did not last for 24 h; however, water intake increased significantly during the first 24 hs. These results indicate that inactivation of SHy GABA_A_ receptors is sufficient to elicit feeding and water drinking, and that GABA_A_ receptors in the SHy may be a part of the neural circuit of feeding and drinking.

#### 3.2.2 Picrotoxin in the SHy elicits feeding

**Figure 4:**
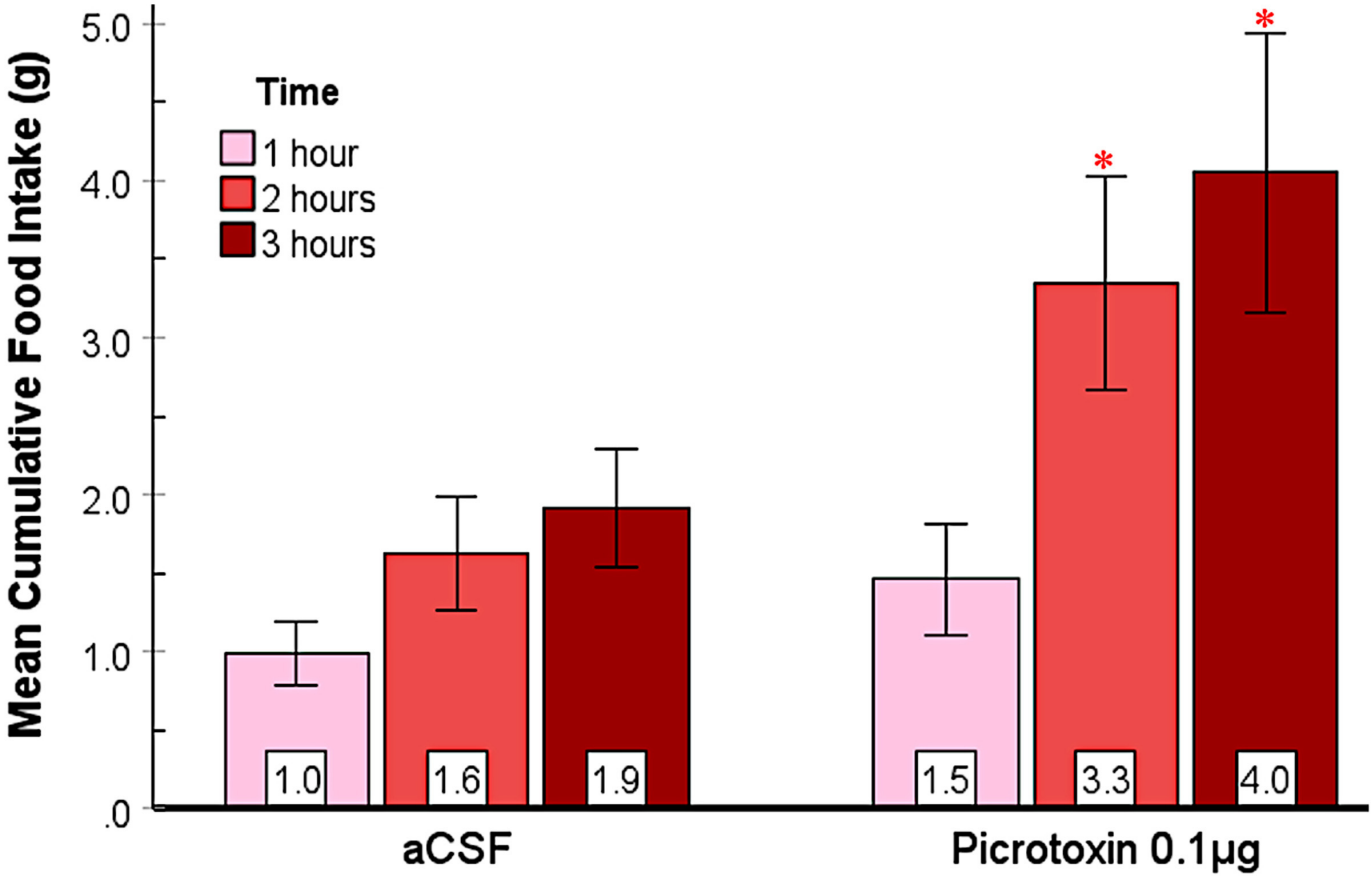
Picrotoxin injected into the SHy elicited a delayed feeding response. Error bars SEM; *n* = 12; mean values are shown at the bottom of each bar. * indicates significant differences compared to the vehicle group at matched times at *p* < 0.05.

The effect of picrotoxin, another GABA_A_ receptor antagonist, on food and water intake in the SHy was examined. A 2-way repeated measures ANOVA (*n* = 12; Figure 4) showed significant main effects for both drugs [*F*_(1, 11)_ = 5.81, *p* = 0.03, $\eta_{p}^{2}=0.35$] and time [*F*_(1, 11.4)_ = 14.80, *p* < 0.01, $\eta_{p}^{2}=0.57$], and significant interaction [*F*_(1.1, 12.2)_ = 5.17, *p* = 0.04, $\eta_{p}^{2}=0.32$] between drugs and time. Based on the *post hoc* analysis, picrotoxin elicited significantly more feeding at 2 (*p* = 0.03) and 3 (*p* = 0.03) hours post-injection but not during the first hour, suggesting that picrotoxin is sufficient to induce feeding in rats. Mean individual food intakes at 3 h are indicated in red in Figures 2b–e. The 24 h measurements did not reveal statistically significant differences (Supplementary Figure S1), suggesting that picrotoxin did not have long-term effects on feeding. Water drinking analysis revealed significant main effects for drugs [*F*_(1, 11)_ = 11.16, *p* < 0.01, $\eta_{p}^{2}=0.50$] and time [*F*_(1.2, 13.2)_ = 249.37, *p* < 0.001, $\eta_{p}^{2}=0.96$], and a significant interaction [*F*_(1.1, 12.2)_ = 8.74, *p* = 0.01, $\eta_{p}^{2}=0.44$]. Based on the *post hoc* analysis, the picrotoxin group drank more water compared to controls after the 2nd (*p* < 0.01) and the 3rd (*p* < 0.01) hours post-injection (Supplementary Figure S3), which coincided with the feeding effect. However, by the 24-h measurement, there were no significant differences in water drinking (figure not shown). This data provides converging evidence that SHy GABA_A_ receptor inactivation is sufficient to elicit feeding and drinking in rats.

#### 3.2.3 2-OH saclofen rapidly elicits feeding in the SHy

**Figure 5:**
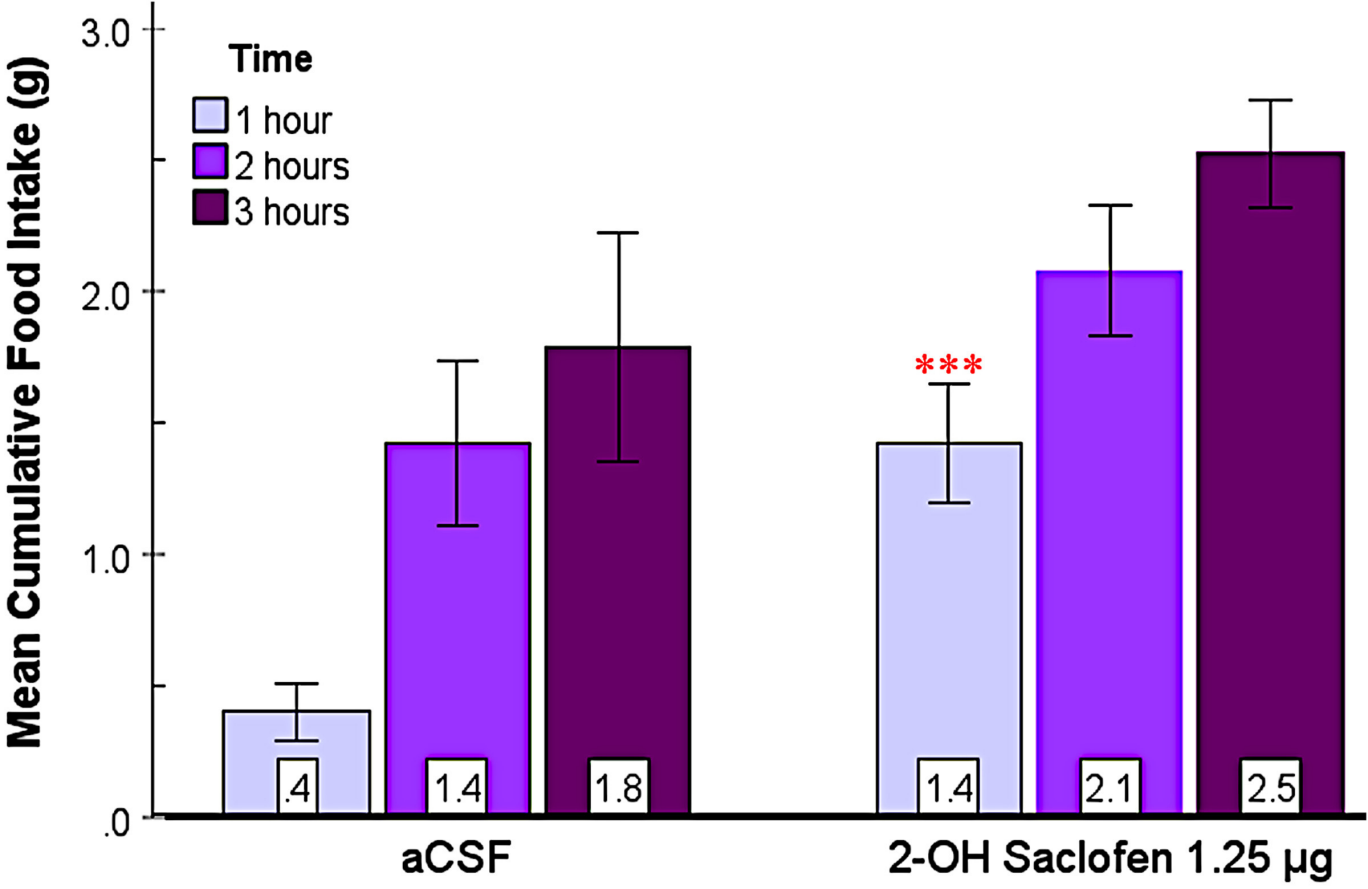
2-OH saclofen injected into the SHy elicited a rapid feeding response. Error bars SEM; *n* = 10; mean values are shown at the bottom of each bar. *** indicates a significant main effect for the 2-OH Saclofen group compared to vehicles at matched times at *p* < 0.001.

This experiment aimed to assess the effect of 2-OH saclofen, a GABA_B_ receptor antagonist, on food and water intake in the SHy. As shown in Figure 5, 2-OH saclofen rapidly elicited feeding in rats during the first hour. The 2-way repeated measures ANOVA (*n* = 10) showed significant main effects for drug injection [*F*_(1, 9)_ = 10.08, *p* = 0.01, $\eta_{p}^{2}=0.53$] and time [*F*_(2, 18)_ = 49.58, *p* < 0.001, $\eta_{p}^{2}=0.85$]. Based on the *post hoc* analysis, the 2-OH saclofen group ate significantly more food than controls 1 h post-injection (*p* < 0.001; Figure 5). Mean individual food intakes at 3 h are shown in purple in Figures 2b–e. The interaction of time and drugs was not significant. These results indicate that 2-OH saclofen rapidly elicited a feeding response post-injection (2-OH saclofen rat eating.mov). Similarly to the GABA_A_ antagonists, the 24-h food intake measurements post 2-OH saclofen injection did not reveal statistically significant differences (Supplementary Figure S1). Based on these non-significant results, neither GABA_A_ nor GABA_B_ antagonists affected feeding in the long term. In addition, the analysis of water intake only showed significant main effects for time, but not for drugs or the interaction during the first 3 hs, suggesting that 2-OH saclofen injection did not modulate water drinking. These results suggest that the inactivation of SHy GABA_B_ receptors is sufficient to rapidly elicit feeding, but not water drinking. This experiment provides evidence that the SHy GABA_B_ receptors may also be a part of the neural feeding circuit.

#### 3.2.4 No increase in feeding from off-target injections

**Figure 6:**
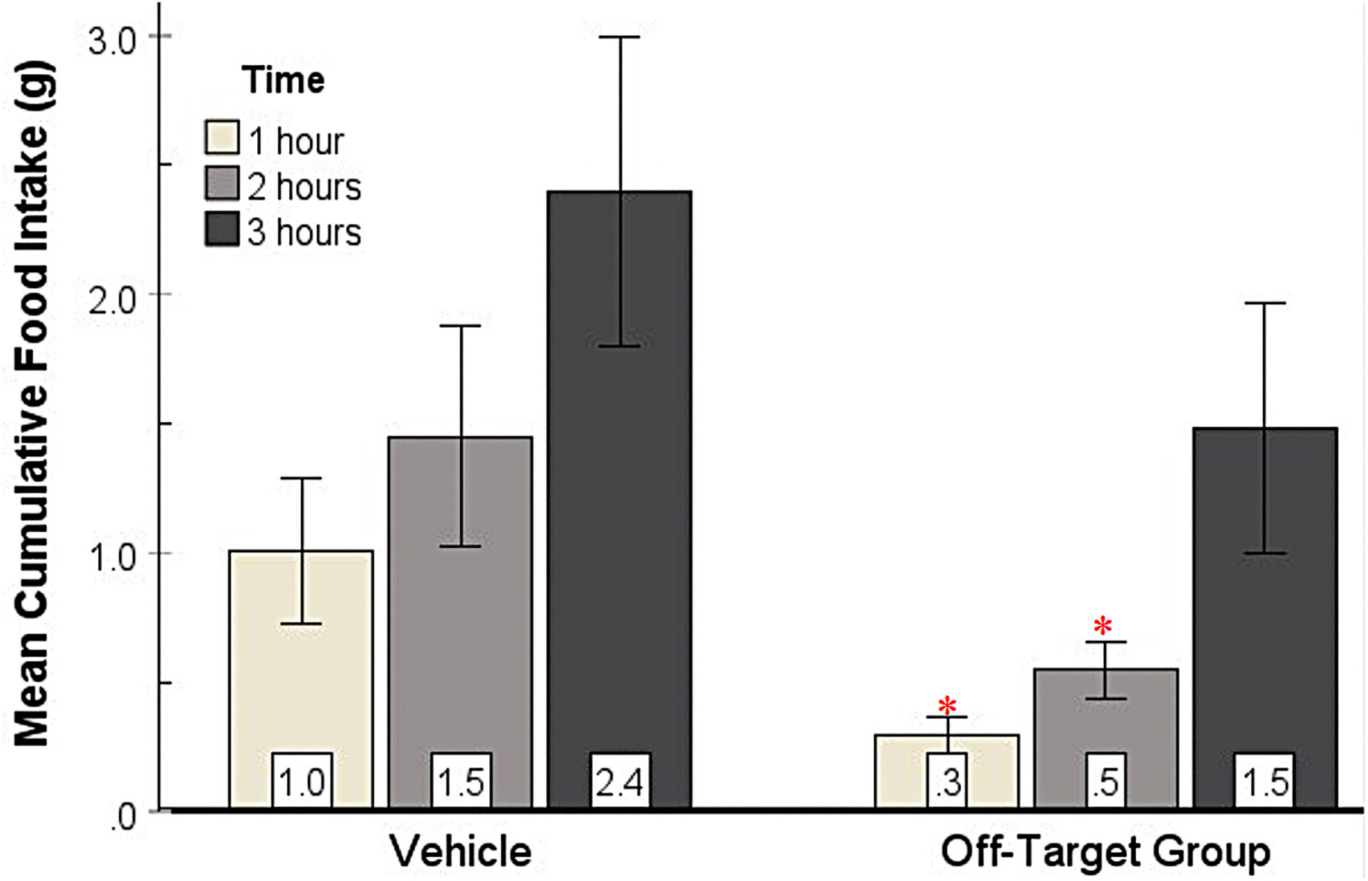
Food intake of Off-target injections compared to vehicle. Food intake was not elicited by injections of GABA antagonists in the areas surrounding the SHy. Error bars SEM; *n* = 8; mean values are shown at the bottom of each bar. * indicates a significant main effect for the 2-OH Saclofen group compared to vehicles at matched times at *p* < 0.05.

To provide an anatomical control for feeding produced by injections within the SHy, we created an Off-target group (*n* = 8) from subjects with histologically determined injection sites outside of the SHy. Based on the 2-way repeated measures ANOVA, there was a significant main effect for drug injection [*F*_(1, 7)_ = 7.12, *p* = 0.03, $\eta_{p}^{2}=0.50$] and time [*F*_(1.2, 8.4)_ = 7.31, *p* = 0.02, $\eta_{p}^{2}=0.51$], but there was no significant interaction (Figure 6). *Post hoc* analysis showed that food intake was actually significantly less in the drug injection group in both the first hour (*p* = 0.04) and second hour (*p* = 0.04) post injection. Mean individual food intakes at 3 h are shown outside of the target site in Figures 2b–e. The 24-h measurements did not reveal statistically significant differences in food intake (Supplementary Figure S1). In addition, water intake was also not significantly different (Figure not shown). These results show that injections of GABA receptor antagonists in the areas surrounding the SHy do not elicit feeding. In fact, GABA antagonists have been shown to reduce feeding in the LS, an area adjacent to the SHy (Gabriella et al. (2022)), which may explain the reason why food intake appears to be reduced in the drug group compared to the vehicle group.

### 3.3 Behavioral results

Multiple behaviors were evaluated with minute-by-minute visual observation for one h post GABA_A_ and GABA_B_ receptor antagonist injection in the SHy. To assess the behavioral results, the data from all three experiments were compared to each other by the behaviors. The data were cumulatively calculated at 30 and 60 min to examine immediate responses versus delayed responses. The vehicle injection data from each experiment were pooled together to create one vehicle group and randomly sampled to have a relatively equal sample size (*n* = 12). A Mixed Model ANOVA was employed to analyze the behavioral data, followed by a pairwise comparison *post hoc* test. Videos of rats can be viewed in our Supplementary materials at the Open Science Framework website (Gabriella (2025b).

**Figure 7:**
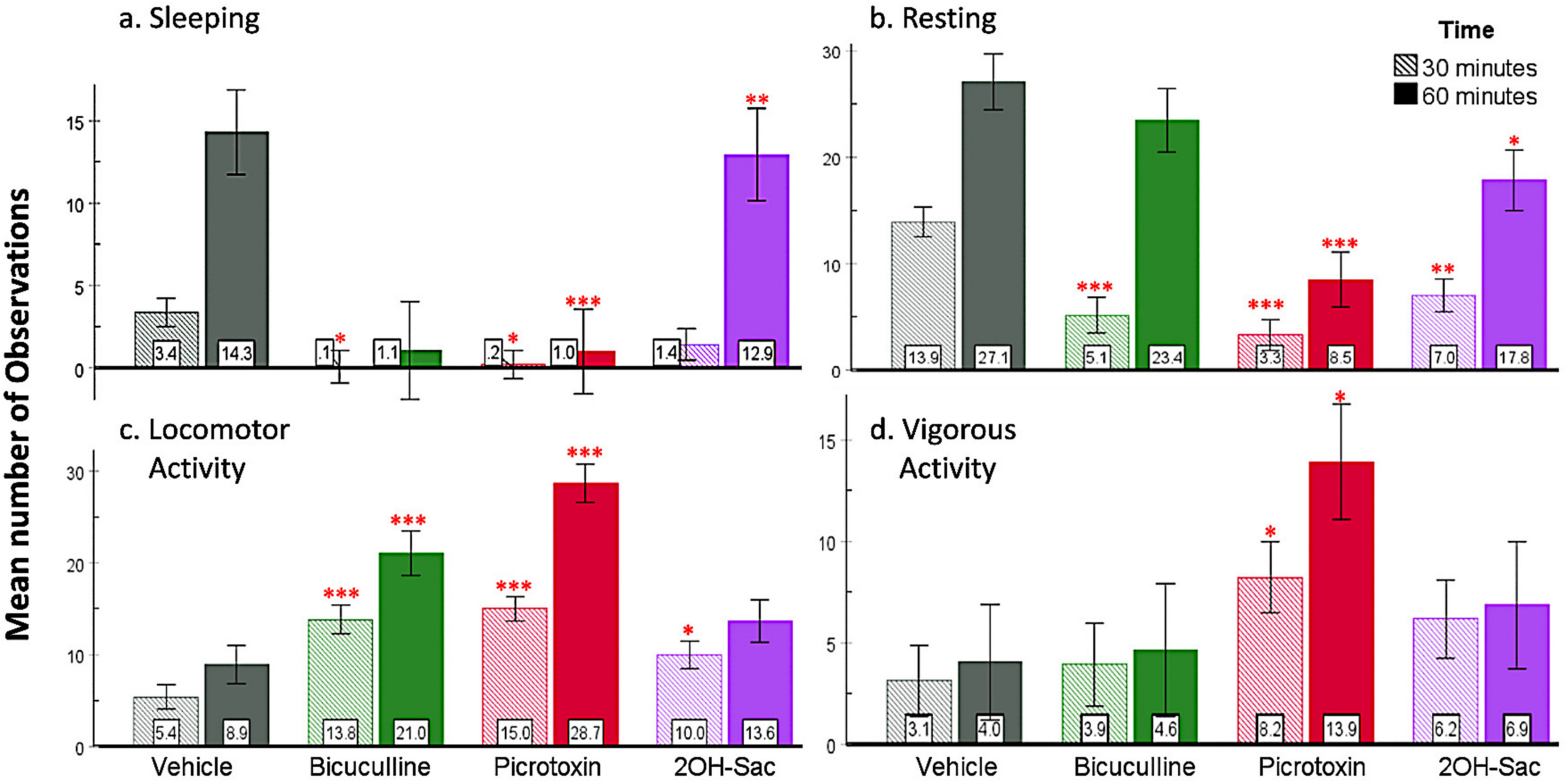
Behavioral results based on number of observations. **(a)** Picrotoxin and bicuculline injected into the SHy suppressed sleeping. **(b)** During the first 30 min, all three drug injections suppressed resting, while only picrotoxin and 2OH-Saclofen continued to suppress resting during the first hour. **(c)** Locomotor activity increased in all three groups (bicuculline, picrotoxin, and 2-OH Saclofen) compared to vehicles during the first 30 min and remained significant in the bicuculline and picrotoxin groups at 60 min. **(d)** Vigorous activity only increased in the picrotoxin group. Error bars SEM; mean values are shown at the bottom of each bar. *** indicates significant differences compared to the vehicle group at matched times at *p* < 0.001. Similarly, ** indicates *p* < 0.01, and * indicates *p* < 0.05. Green = Bicuculline; Red = Picrotoxin; Purple = 2-OH Saclofen.

Sleeping was observed for 1 h post-injection in all rats. Mixed ANOVA showed a significant interaction of time and drug [*F*_(3, 39)_ = 7.42, *p* < 0.001, $\eta_{p}^{2}=0.36$; Figure 7a]. Based on the *post hoc* analyses, bicuculline (*p* = 0.02) and picrotoxin (*p* = 0.01) suppressed sleeping compared to the control group at 30 min and at 60 min (bicuculline: *p* < 0.01; picrotoxin: *p* < 0.001). These results indicate that picrotoxin and bicuculline injections suppressed sleeping in rats throughout the first hour, while 2-OH-Saclofen did not.

We observed resting in all rats for one h after the injections. Based on the analysis, drug and time had a significant interaction [*F*_(3, 39)_ = 8.53, *p* < 0.001, $\eta_{p}^{2}=0.39$; Figure 7b]. *Post hoc* analysis revealed that all three drugs suppressed resting compared to the control group (bicuculline *p* < 0.001, picrotoxin *p* < 0.001, and 2-OH saclofen *p* < 0.01) during the first 30 minutes postinjection. At the 60-minute measurement, only picrotoxin (*p* < 0.001) and 2-OH saclofen (*p* = 0.2) suppressed resting. Based on these results, all three drug injections rapidly suppressed resting for at least 30 minutes after injection (Supplementary materials: Picro1.mov, Bic 1.mov, 2-OH Sac 1.mov, and aCSF 1.mov).

Locomotor activity (being upright, light movement) was also measured for 1 h after the injection. The analysis revealed that there was a significant interaction between drug and time [*F*_(3, 39)_ = 18.47, *p* < 0.001, $\eta_{p}^{2}=0.59$; Figure 7c]. *Post hoc* analysis showed that locomotor activity increased due to all three drugs (bicuculline *p* < 0.001, picrotoxin *p* < 0.001, and 2-OH saclofen *p* = 0.02) at 30 minutes. At 60 minutes, the bicuculline (*p* < 0.001) and the picrotoxin (*p* < 0.001) groups were still exhibiting increased locomotor activity compared to controls, but the 2-OH saclofen group did not. These results suggest that all three drugs increased locomotor activity in rats compared to controls, especially picrotoxin and bicuculline.

We also measured vigorous activity (walking up and down, rearing) in rats for the first hour post-injection. A significant interaction [*F*_(3, 39)_ = 4.40, *p* < 0.01, $\eta_{p}^{2}=0.25$, Figure 7d] was found between drug and time. Posthoc analysis revealed that only picrotoxin increased vigorous activity in rats (*p* = 0.05) during the first 30 and 60 min (*p* = 0.02; Supplementary materials: Picro 2.mov). The other groups were not significantly different from the vehicles. Based on these results, only picrotoxin injection elevated vigorous activity compared to controls.

Grooming behavior was also observed during the first hour post-injection. The analysis revealed a significant interaction between time and drug treatment, *F*_(_3, 39) = 3.75, *p* = 0.02, $\eta_{p}^{2}=0.22$ (Supplementary Figure S4). *Post hoc* comparisons indicated that grooming was significantly increased by bicuculline during both the first 30 minutes (*p* = 0.02) and at 60 minutes (*p* = 0.02), relative to control animals. However, picrotoxin or 2-OH saclofen did not modulate grooming.

## 4 Discussion

This study aimed to reveal whether the SHy has a role in modulating eating. We used central microinjections of GABA_A_ and GABA_B_ receptor antagonists to examine their effects on food and water intake and several other behaviors. We hypothesized that the SHy contains GABA receptors and that antagonizing these receptors would induce feeding. Our main finding is that all three antagonists, bicuculline, picrotoxin, and 2-OH Saclofen, elicited statistically significant feeding in rats compared to controls (Figures 3–5, respectively), and bicuculline and picrotoxin also modulated water drinking to a smaller degree. More specifically, our novel findings suggest that SHy neurons and GABA_A_ and GABA_B_ receptors within this nucleus may be part of a neurocircuit involved in the control of eating behavior. These findings are consistent with previous research suggesting that neural activity in this particular brain region is linked to changes in feeding status (Csikós et al., 2020; Kakall et al., 2021; Nakahara et al., 2004; Stratford, 2005). Additionally, we examined the general behavioral effects of these antagonists in the SHy. We found that GABA_A_ and GABA_B_ antagonists (bicuculline, picrotoxin, and 2-OH Saclofen) decreased sleeping and resting while increasing locomotor activity (Figure 7). These findings suggest that GABA receptors on SHy neurons may have functions other than or in addition to the modulation of feeding.

All three GABA antagonists elicited feeding with variously delayed effects. The GABA_B_ antagonist showed relatively rapid results within one h, while the GABA_A_ antagonists were delayed for over one h after injection. The temporal differences between the electrophysiological effects of GABA_A_ and GABA_B_ receptors are in the range of milliseconds, therefore, these processes are unlikely to be due to the differences in feeding between the two receptor types. While delayed feeding effects are common and are similar to other studies investigating nearby brain regions, such as morphine in the lateral septum (Calderwood et al., 2020), opioids in the paraventricular nucleus and other hypothalamic areas (Stanley et al., 1988), they indicate that the GABA antagonists did not have an immediate effect on feeding behavior, even if it rapidly impacted neural activity. It is likely that GABA receptors aren't the only ones in the SHy involved in feeding and may exert their action through a mechanism involving other receptor types. Najimi et al. (2014) found neurotensin receptors, and Mansour et al. (1994) found mu opioid receptors in the SHy. In addition, oxytocin and vasopressin receptors have also been localized here (Jirikowski et al., 1988; Rogers Flattery et al., 2021). These receptors in the SHy may be involved in feeding and could potentially have a more rapid effect than the GABA receptor types. The exact mechanisms underlying the delayed responses in both the GABA_A_ and GABA_B_ receptors are not known.

Although modest in magnitude, we also observed an increase in water intake following GABA_A_, but not GABA_B_ receptor antagonism. This finding is consistent with previous work by Houston et al. (2002), who demonstrated that subcutaneous administration of bicuculline reversed the suppressive effects of muscimol on water intake in rats. Although the precise role of the SHy in regulating fluid consumption remains unclear, these results support the notion that GABAergic inhibition contributes to the modulation of water intake. Specifically, antagonism of GABA_A_ receptors may relieve tonic inhibitory control, thereby facilitating water-seeking behavior even in the absence of a strong homeostatic drive.

The potential spread of the GABA antagonists into other brain regions may bring the anatomical specificity of these results into question. However, the diffusion of dye and other neurotransmitters from previous intracerebral microinjection studies support local actions for our GABA antagonist injections. For example, (Myers, 1966) found that 0.5 μl dye injected into the diencephalon only diffused about 0.3–0.8 mm from the injection site. In addition, a volume of 1.0 microliter dye still only spread 1.1 mm into the surrounding tissue. Myers et al. (1971) also showed that acetylcholine (ACh 8 mg/ml), serotonin (5-HT 2.5 mg/ml), and norepinephrine (NE 2.0 mg/ml) injected into the hypothalamus at a 1 μl volume through a chronically fixed cannula mainly stayed in the hypothalamus, with less than 1% leakage into the ventricles from ACh and 5-HT, and less than 2% of the injected volume of NE. In another study, Myers and Hoch (1978) injected dopamine (3.01 μg/μl) into the pars compacta of the substantia nigra at different volumes (0.5, 1.0, 4.0, and 8.0 μl) and showed that while the magnitude of dispersion was proportional to the volume injected, the concentration had a ceiling effect, which suggested a limit on the solution's potential saturation. These studies together reveal that the possible dispersion of injected solutions is limited and commensurate with the volume. In the current study, the volume injected into the rats was 0.3 μl, which is a lower volume than what was investigated in these previous studies; therefore, a corollary assumption is that the dispersion was even less, and the solutions mostly stayed within the SHy. Another factor supporting our assumption is that the Off-target injections outside the SHy not only did not elicit eating but actually suppressed eating. Noteworthy is that the SHy borders on the lateral septum, where some of the off-target cannulas were located. We have shown in a previous study that GABA receptor antagonists injected directly into the adjacent lateral septum not only do not elicit eating but, unlike the effect in the SHy, it actually suppress feeding (Gabriella et al., 2022). These findings produce a convergence of evidence for GABA antagonists producing feeding specifically in the SHy and not in another brain region due to spread.

Our second aim was to investigate the general behavioral responses, including sleeping, resting, locomotor activity, vigorous activity, grooming, eating, and drinking to the three GABA antagonists in the SHy. Behavioral observations were collected for the first hour post-injection. Our data demonstrates that GABA_A_ and GABA_B_ antagonism decreased sedentary behavior (sleeping, resting) and increased general activity (locomotor and vigorous activity). In particular, picrotoxin and bicuculline decreased sleeping, while all three drugs decreased resting. Bicuculline and picrotoxin increased locomotor activity for 60 min, while 2-OH saclofen only increased it for 30 min. Bicuculline also increased grooming. These results reveal a robust increase in arousal due to GABA_A_ receptor antagonism and a moderate level of arousal due to GABA_B_ receptor antagonism.

The activity observed was only vigorous in the picrotoxin group, not in the bicuculline or the 2-OH Saclofen groups. Whether the greater effectiveness of picrotoxin is due to differences in the properties of the GABA_A_ and GABA_B_ receptors or of the drugs is an open question. Still, it may be noteworthy that picrotoxin is a Cl^-^ channel blocker. As a result, picrotoxin may not only prevent the activation of GABA_A_ receptors but also impedes chloride inflow for any other reason, thereby preventing cell hyperpolarization. Consequently, neurons may become more susceptible to depolarization, leading to heightened overall activity. This postulation may explain why picrotoxin's effects were more pronounced than bicuculline.

Our behavioral results align with previous research. This brain region has been implicated in negative emotional stimuli, such as stress (Campeau et al., 1997), fear (Day et al., 2004), and social defeat (Nasanbuyan et al., 2025). Thus, unsurprisingly, in our study, inhibiting SHy GABAergic neurons leads to increased general arousal and activity, such as moving up and down, rearing on hind legs, or grooming. These activities may be associated with emotional stimuli similar to the stress response; it could be escape behavior or food seeking, and grooming may be a sign of self-soothing in times of stress (Lim and Hong, 2023).

In many cases, both locomotion and hyperphagia can be related to stress (Patterson and Abizaid, 2013). The hypothalamic-pituitary-adrenal response to perceived threat in a stressful situation results in glucocorticoid production. Glucocorticoids can affect metabolism and can increase food-seeking behavior and locomotor activity (Patterson and Abizaid, 2013). For example, a tail pinch is a mild, non-specific type of stress that has been shown to induce hyperphagia (Antelman et al., 1976). The feeding behavior is especially pronounced when the available food is highly palatable, as in our study. In addition, tail pinch and other mildly stressful stimulations can induce other stress-related behaviors, such as maternal behavior, copulatory behavior, or grooming (Rowland and Antelman, 1976). Therefore, it is possible that the SHy brain region is involved in a type of stress response. Because the overall effect of the GABA_B_ receptor antagonist, 2-OH saclofen, on behavioral arousal was much less pronounced than the GABA_A_ antagonists, this may be an indication that GABA_B_ receptors in the SHy are less involved in these emotional responses to stressful stimuli. The specific mechanism of the behavior activated by SHy GABA receptor antagonism is not currently known.

Despite the SHy's potential involvement in the stress response, the aggregate of our results indicates that the feeding response may be independent of the behavioral response. The GABA_B_ antagonist group responded to the injection with rapid feeding during the first hour. This rapid feeding happened simultaneously with the increased activity. Therefore, at least in the GABA_B_ group, feeding could not be simply a consequence of becoming tired from moving around and needing to replenish resources. In addition, the activity observed was only vigorous in the picrotoxin group, not in the bicuculline or 2-OH saclofen groups. Yet, this study showed that feeding was robustly elicited in all three antagonist groups despite the varying levels of general arousal. This points to a specific role for SHy GABA receptors in feeding, independent of the observed increases in other behaviors.

The postulation that the SHy is specifically involved in feeding is further supported by its connection to eating-related brain regions. The SHy is surrounded by and connected with other brain regions that are important in the feeding circuit. Although our results don't indicate that the SHy may be a prominent center for feeding, we provide evidence of its role within the feeding circuit. Previous research has shown connections to multiple areas of the hypothalamus (Barbier et al., 2021; Chiba and Murata, 1985; Nasanbuyan et al., 2025; Ugur et al., 2021) and the septum (Jirikowski et al., 1988; Shin and Ikemoto, 2010; Yeates et al., 2022), both of which are adjacent and are involved in feeding. A corollary assumption is that the SHy brain region may be a part of the septohypothalamic circuit. More specifically, it may be an indirect connection between the lateral septum and other nuclei of the hypothalamus. In addition, the SHy was found to be activated after muscimol injection in the nucleus accumbens (Stratford, 2005), which is associated with induced feeding. Therefore, it is also possible that the SHy indirectly connects the nucleus accumbens shell, the lateral septum, and several nuclei of the hypothalamus, providing an alternative route to transmit feeding signals. It is notable to mention that the nucleus of the solitary tract sends projections to the SHy (Zhu et al., 2024). The nucleus of the solitary tract processes vagal afferent signals related to ingestion (Grill and Hayes, 2009), cranial nerve signals related to the mouth and chewing (facial, glossopharyngeal, and trigeminal nerves), and disseminates this information to various feeding-related brain regions (Bradley, 2006). The projection from the nucleus of the solitary tract to the SHy further supports its role in the feeding circuit.

While our study only involved male rats, it is important to highlight the potential for sexually dimorphic results. Male rats are known to eat more and have less body fat than female rats, and these differences can not be explained by body mass or metabolic rate (Asarian and Geary, 2013). Instead, both organizational and activational effects of estrogen and androgen contribute to these differences. For example, neonatal testosterone reduces pro-opiomelanocortin neuronal fibers and projections in the arcuate nucleus of the hypothalamus in male rats, but not in females, which results in increased food intake in males. In addition, circulating androgen levels decrease leptin in males, but estrogen levels increase leptin in females, further contributing to more feeding in males (Haque and Tischkau, 2022). In rats, gonadotropin-releasing hormone, and kisspeptin neurons are stimulated by estrogen in the preoptic hypothalamic area, which is adjacent to the SHy. Based on the potential connections of the SHy to hypothalamic brain regions involved in these processes, it is possible that our findings are only specific to male rats, and may be moderated in female rats. Our serendipitous observations before the study were made in male Sprague–Dawley rats, and we prioritized replicating and confirming these effects within the same sex and strain to maintain consistency. This approach also facilitated comparisons with the limited number of previous studies referencing the SHy brain region, the majority of which also utilized male rats. However, future research should investigate sex differences in feeding in the SHy.

Given our findings suggesting a role for GABA_A_ receptors in feeding behavior, it is plausible that administration of a GABA_A_ receptor agonist—rather than an antagonist—may suppress feeding in rats. Future studies could also investigate the effects of GABA_A_ agonists in the SHy to further elucidate the functional role of this receptor system in the regulation of feeding. In addition, future research could utilize *in situ* hybridization to verify the specific receptor types present in the SHy, or apply immunohistochemistry or immunofluorescence techniques to assess the relative expression levels of receptors. Behavioral assays may be employed to explore the potential involvement of neurotensin, mu-opioid, oxytocin, and vasopressin receptors, as well as distinct neuronal populations, in the regulation of feeding behavior. Identifying a distinct receptor type within the SHy that modulates feeding without affecting general arousal could help dissociate feeding-specific effects from other motivated behaviors, thereby clarifying the unique contribution of this brain region to the regulation of food intake.

### 4.1 Conclusion

Our results suggest that deactivating GABA_A_ or GABA_B_ receptors with antagonists robustly induced feeding and heightened arousal in rats. Taken together, these findings imply that SHy neurons containing GABA_A_ and/or GABA_B_ receptors are parts of a neural circuit involved in the control of feeding behavior. This study provides a starting point for future research examining feeding or other behavioral responses in the SHy and may have implications for widening scientific knowledge about the septohypothalamic circuit. Better understanding the SHy in the context of a larger feeding circuit may have implications for the current obesity trends, and disordered eating.

## Data Availability

The datasets presented in this study can be found in online repositories. The names of the repository/repositories and accession number(s) can be found in the article/Supplementary material.

## Supplementary material

Supplementary Figure 1 No significant differences in food intake at 24 h. Error bars SEM; mean values are shown at the bottom of each bar.

Supplementary Figure 2 Bicuculline injected into the SHy elicited water drinking. Error bars SEM; *n* = 9; mean values are shown at the bottom of each bar. * indicates significant differences compared to the vehicle group at matched times at *p* < 0.5.

Supplementary Figure 3 Picrotoxin injected into the SHy elicited water drinking. Error bars SEM; *n* = 12; mean values are shown at the bottom of each bar. ** indicates significant differences compared to the vehicle group at matched times at *p* < 0.1.

Supplementary Figure 4 Bicuculline injected into the SHy increased grooming during the first 30 min, and the first hour. Error bars SEM; mean values are shown at the bottom of each bar. * indicates significant differences compared to the vehicle group at matched times at *p* < 0.05.
