# Supplementary material for: GABA receptor antagonism elicits feeding in the septohypothalamic nucleus
Source: Front Behav Neurosci. 2025 Aug 4;19:1633659. doi: 10.3389/fnbeh.2025.1633659 (PMC12358381; doi:10.3389/fnbeh.2025.1633659)
Supplement: Supplementary Figure 1 — No significant differences in food intake at 24 h. Error bars SEM; mean values are shown at the bottom of each bar. [file Presentation_1.pdf]

Supplementary Material

The histology, feeding, and behavioral data sets are available on the Open Science Framework website and can be accessed at https://osf.io/d5kur (Gabriella, 2025a). The website also includes the analysis code.

1 SUPPLEMENTARY TABLES

Vehicle	Mean difference	t	df	p
Food intake at 1 hour	-0.10	-0.72	17	.24
Food intake at 2 hours	-0.49	-1.15	17	.13
Food intake at 3 hours	-0.52	-0.96	17	.17

Table S1. Independent samples t-test did not show significant differences between the DMSO and aCSF food intakes at 1, 2, and 3 hours post injection.

2 SUPPLEMENTARY FIGURES

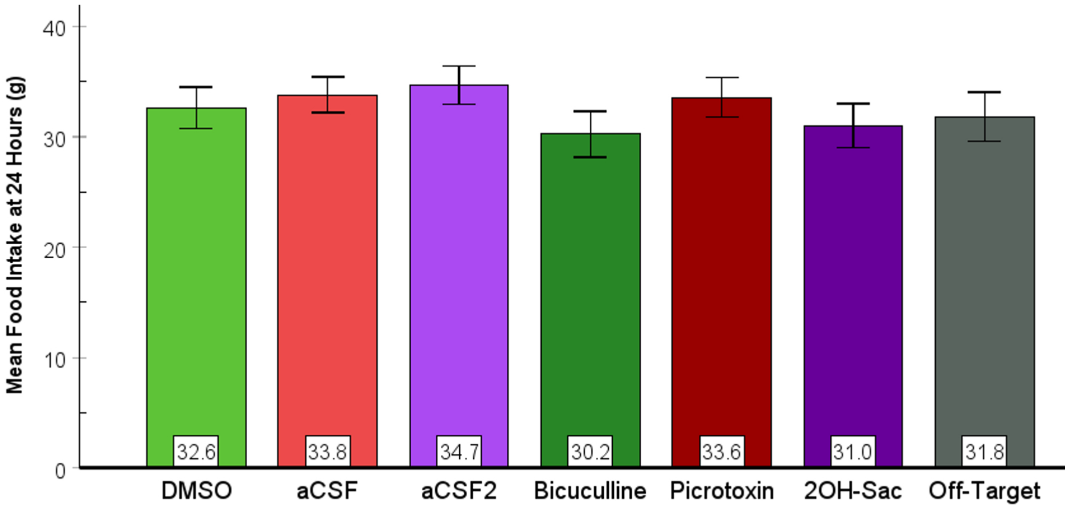

Figure S1. No significant differences in food intake at 24 hours. Error bars SEM; mean values are shown at the bottom of each bar.

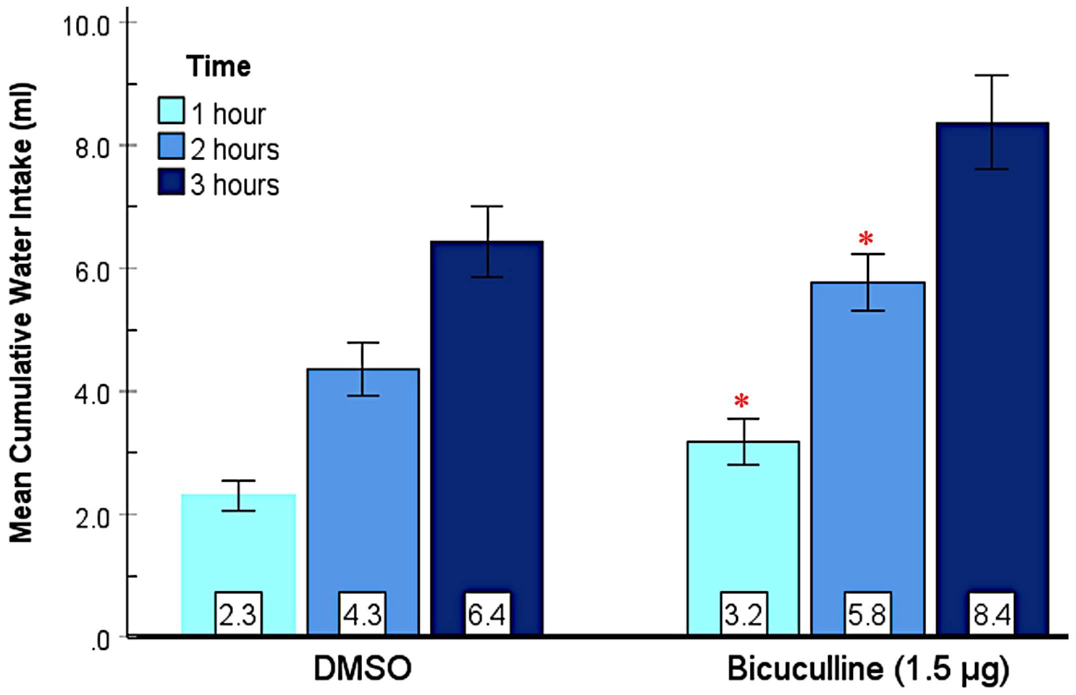

Figure S2. Bicuculline injected into the SHy elicited water drinking. Error bars SEM; n = 9; mean values are shown at the bottom of each bar. * indicates significant differences compared to the vehicle group at matched times at p ¡ 0.5.

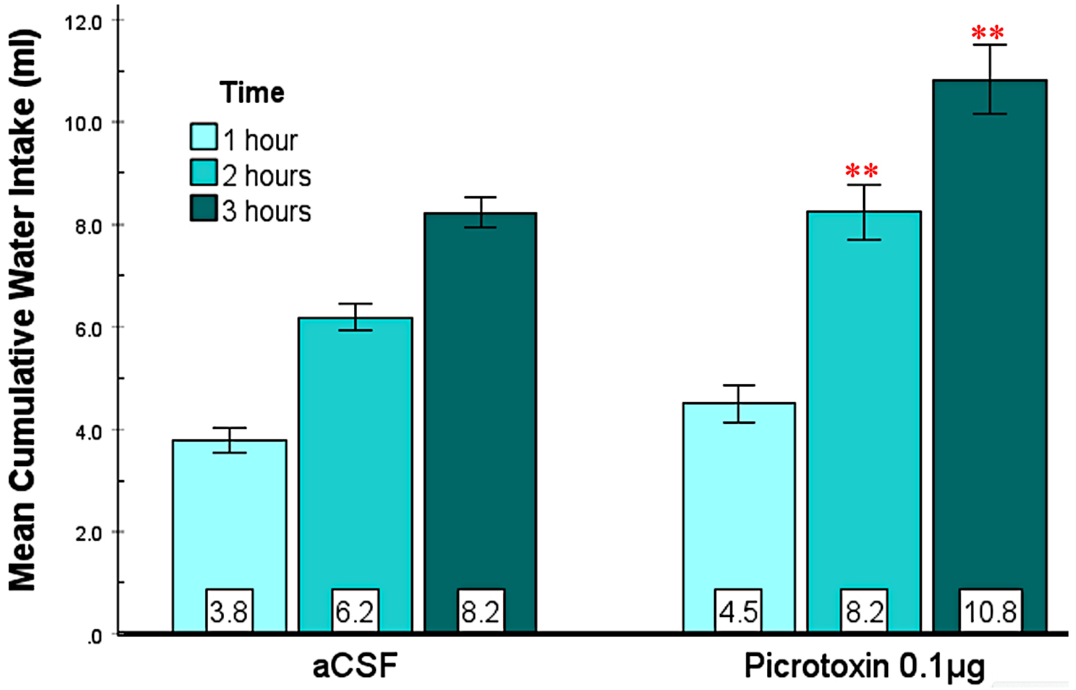

Figure S3. Picrotoxin injected into the SHy elicited water drinking. Error bars SEM; n = 12; mean values are shown at the bottom of each bar. ** indicates significant differences compared to the vehicle group at matched times at p ¡ 0.1.

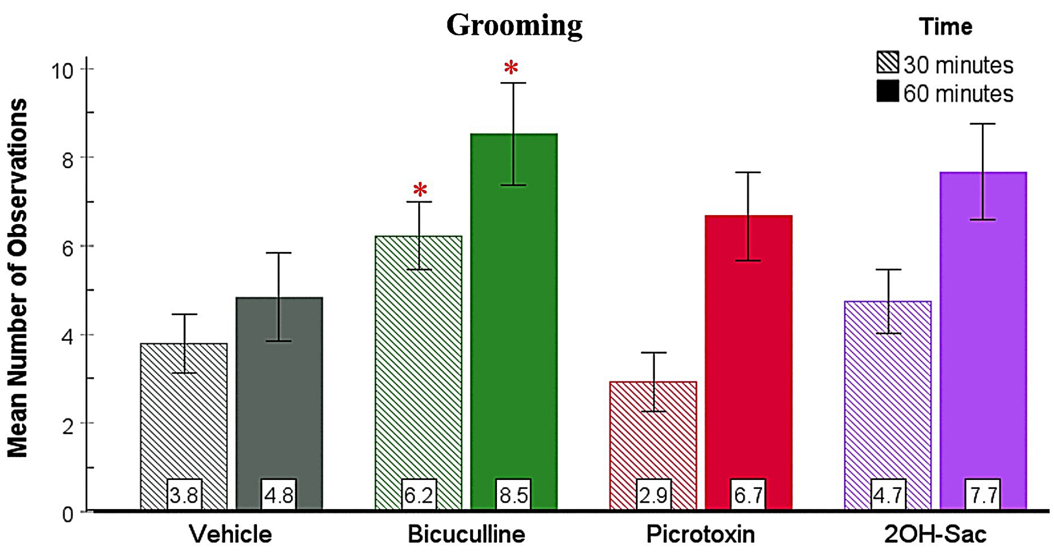

Figure S4. Bicuculline injected into the SHy increased grooming during the first 30 minutes, and the first hour. Error bars SEM; mean values are shown at the bottom of each bar. * indicates significant differences compared to the vehicle group at matched times at p ¡ 0.05.
